# Supplementary material for: Genome-Wide Characterization and Expression Analysis of Fatty acid Desaturase Gene Family in Poplar
Source: Int J Mol Sci. 2022 Sep 21;23(19):11109. doi: 10.3390/ijms231911109 (PMC9570219; doi:10.3390/ijms231911109)
Supplement: Supplementary file 1 [file ijms-23-11109-s001.zip › ijms-1907678-supplementary.pdf]

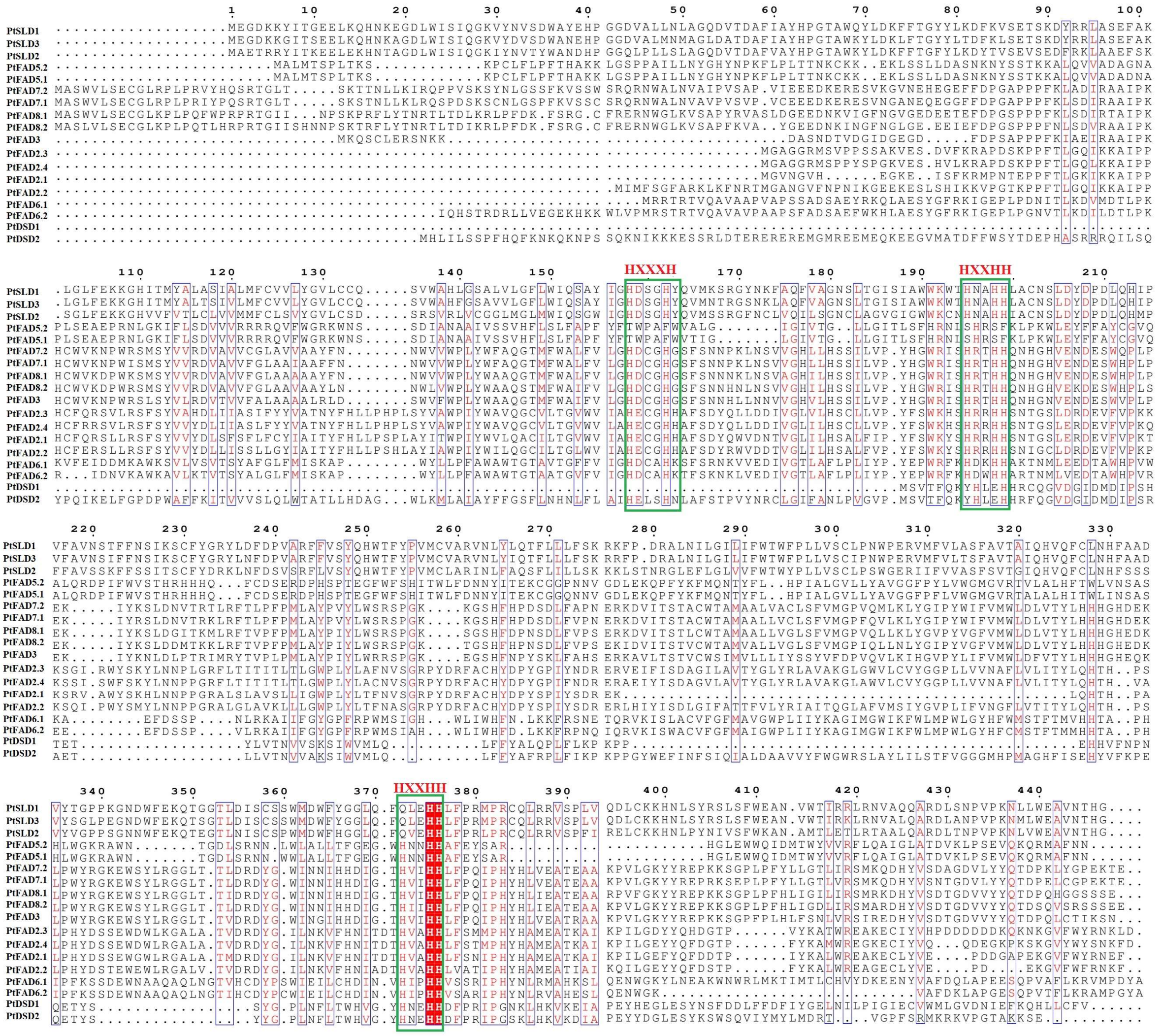

Figure S1. The multiply alignment and conserved domain analysis of PtFAD proteins. The three conserved histidine boxes (HXXXH, HXXHH, and HXXHH) were highlighted in green rectangles.

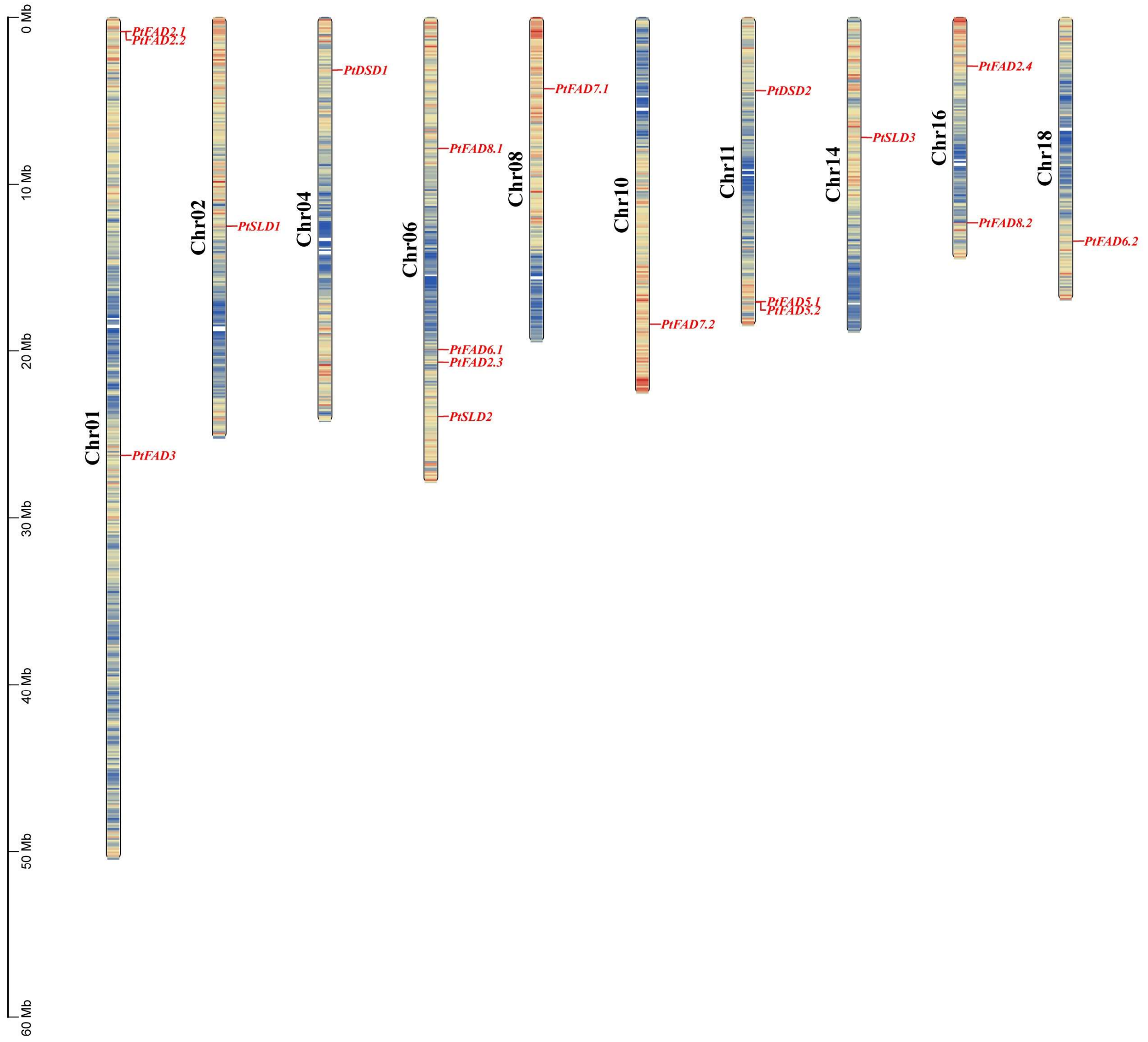

Figure S2. The chromosome distributions of *PtFAD* genes. According to the genome annotation file, the chromosomal locations were displayed from top to bottom on corresponding chromosomes. A total of 23 poplar *FAD* genes were localized on the 12 chromosomes.

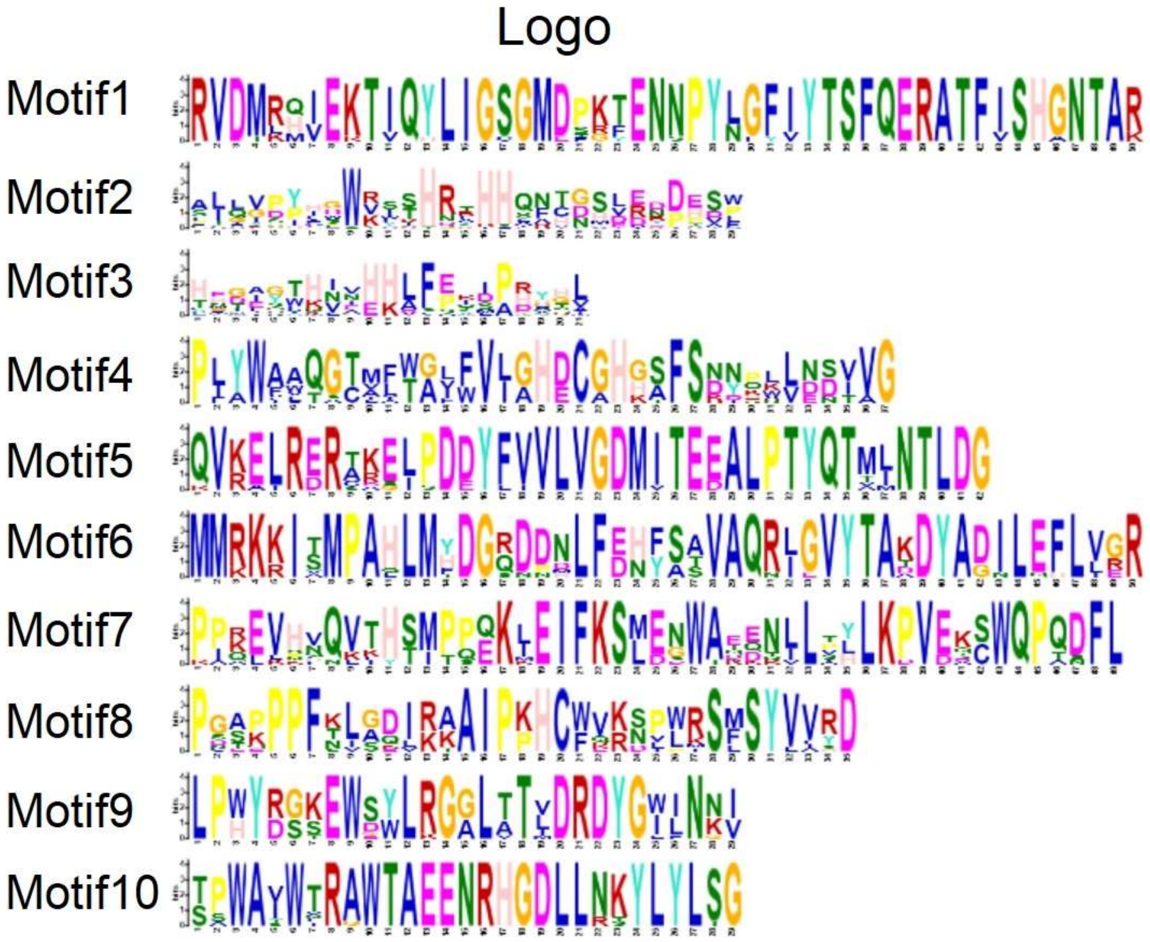

Figure S3. The logo patterns of FAD conserved motifs. The MEME server was performed to analyze conserved motifs of FAD members. The motif1-10 were identified from the FADs, and the number represented the specific motif and component. The different motifs (motif1-10) were represented by differently colored boxes. Amino acid: aa.

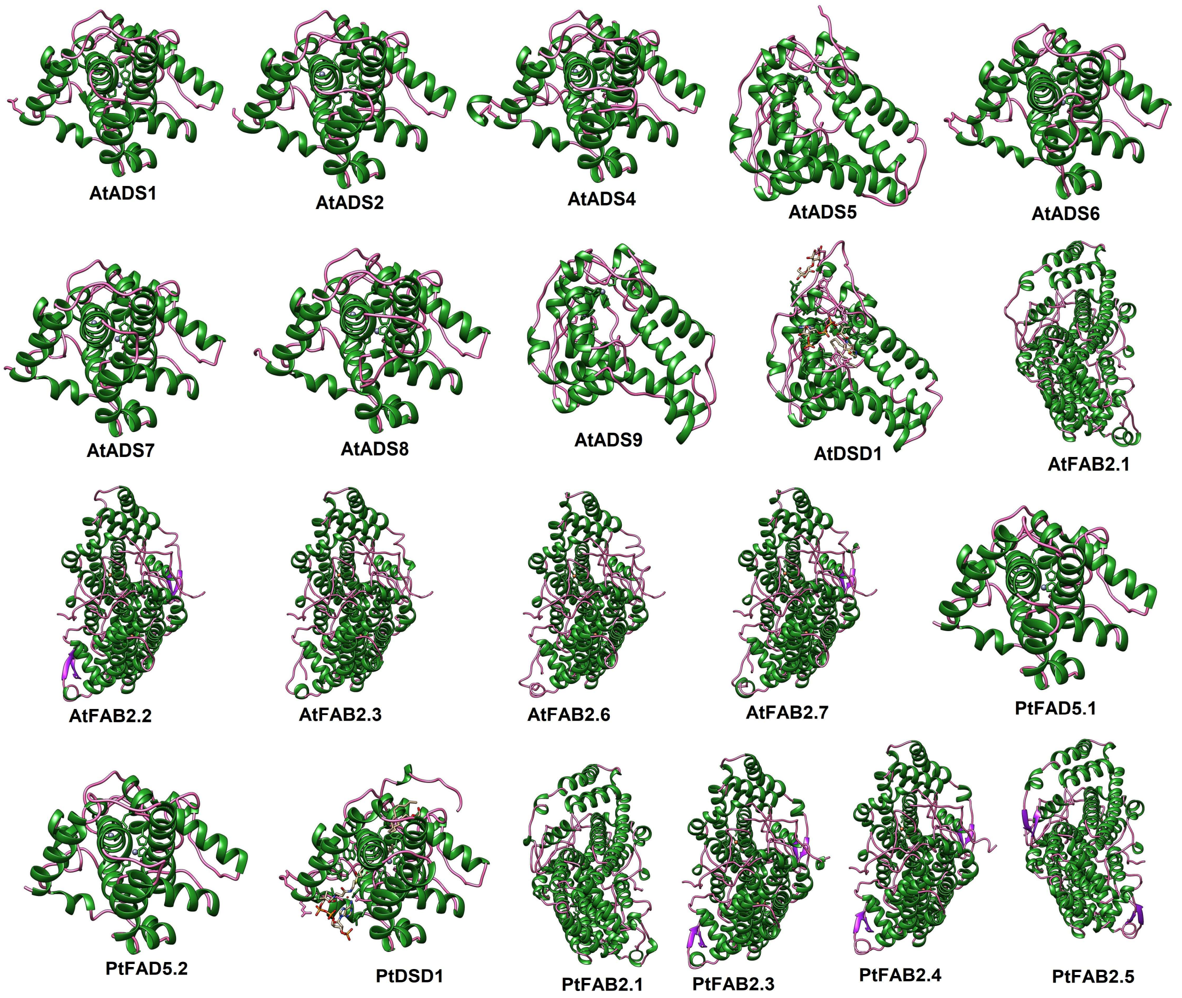

Figure S4. The tertiary structures of AtFAD and PtFAD proteins. The three-dimensional (3D) diagrams of AtFAD and PtFAD were performed in the chimera program. The coil, α-helix, and strand were represented in hot pink, forest green, and purple.

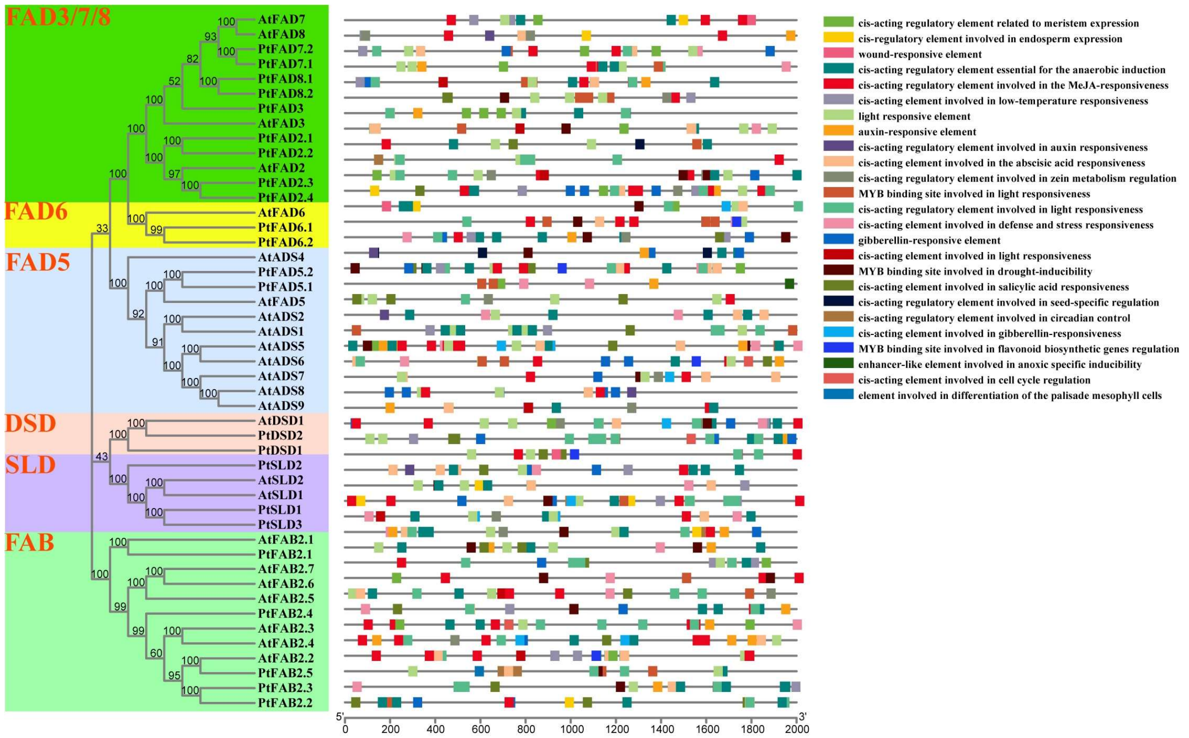

Figure S5. Predicted *cis*-acting elements in the promoters of *FAD* genes. The unrooted phylogenetic tree was shown and constructed using MEGA7.0 set to the neighbor-joining (NJ) method. The different *cis*-acting elements were represented by differently colored boxes, and each group was labeled with a differently colored branch.

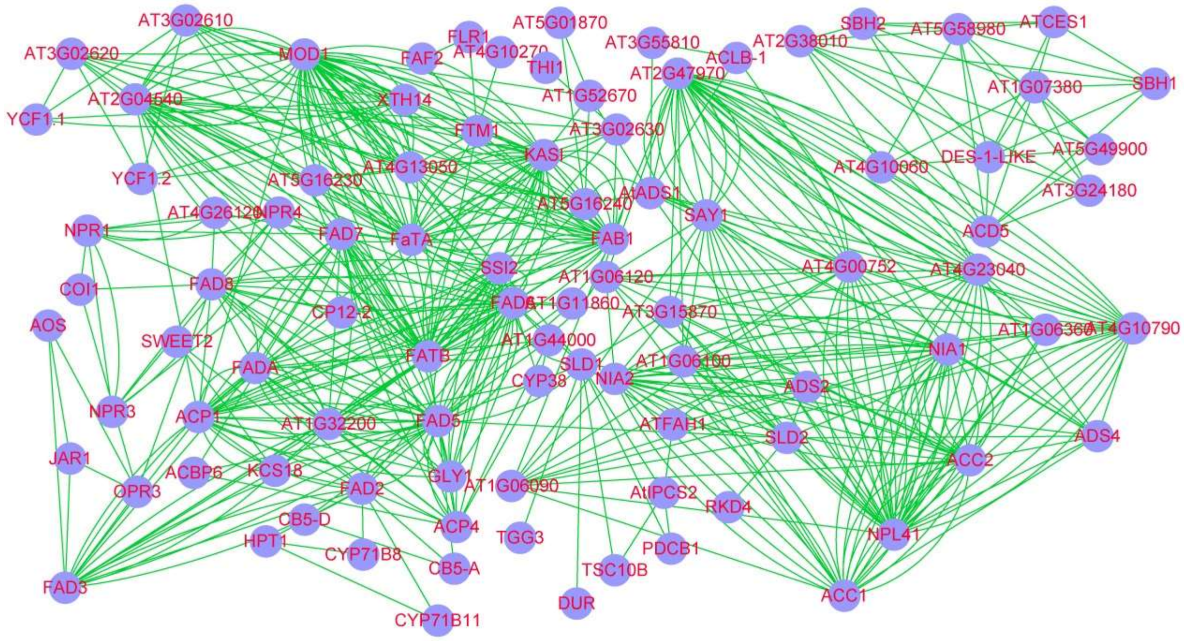

Figure S6. Protein interaction network analysis of the AtFADs. Predicted networks for the AtFADs were generated using the online software String. The green lines indicated the putative interaction relationships, and the blue slate circular indicated interaction proteins.

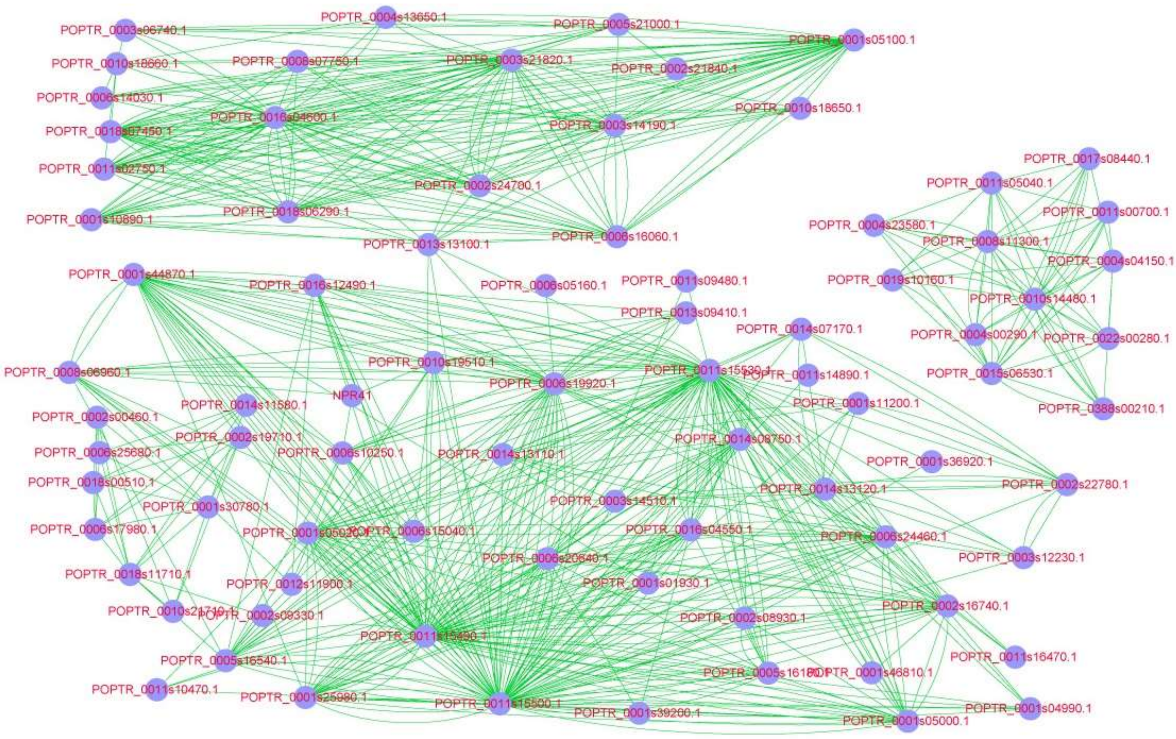

Figure S7. Protein interaction network analysis of PtFADs. Predicted networks for the PtFADs were generated using the online software String. The green lines indicated the putative interaction relationships, and the blue slate circular indicated interaction proteins.

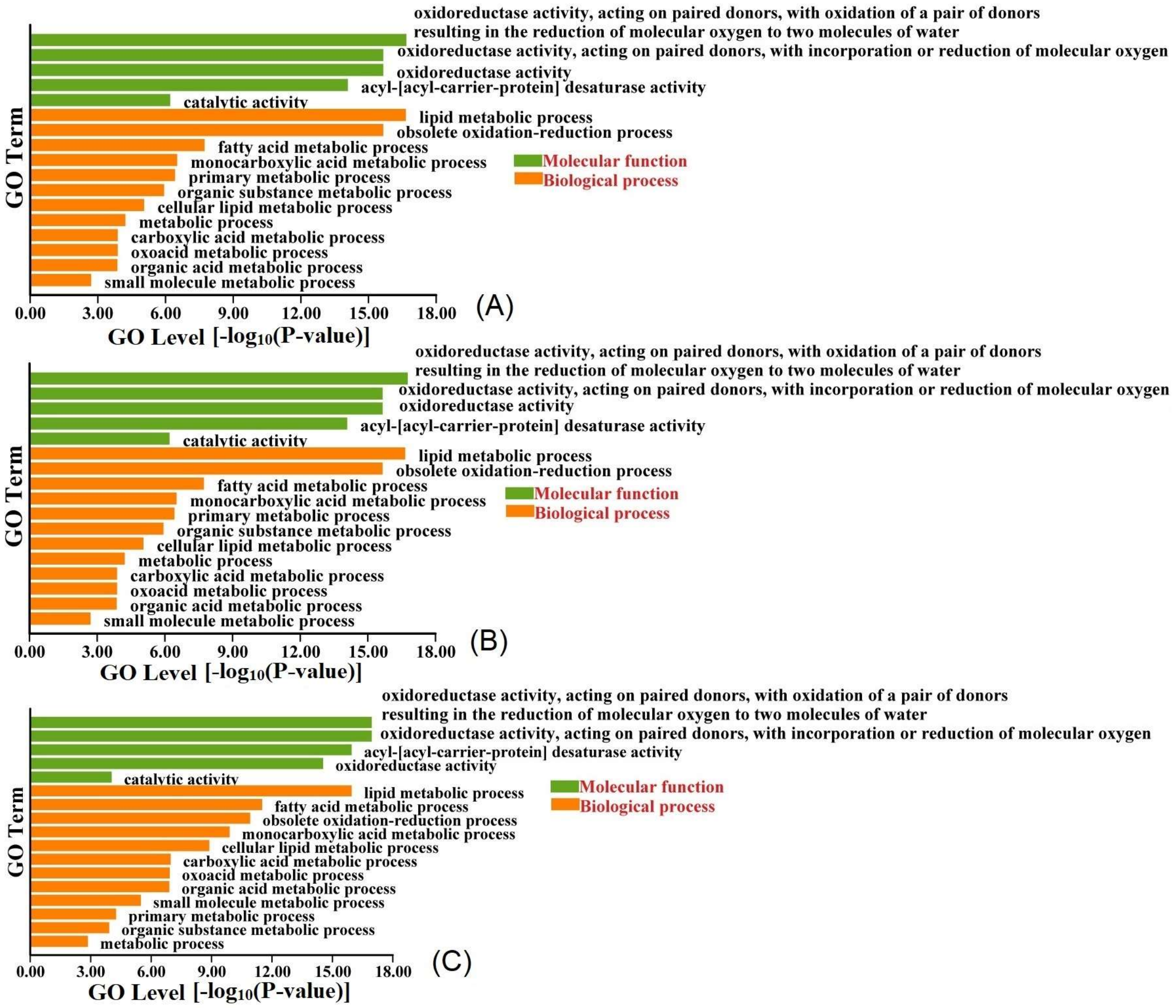

Figure S8. Gene ontology (GO) analysis of *AtFADs* (A), *OsFADs* (B), and *PtFADs* (C), respectively. GO analysis was performed using TBtools with a go-basic file. *AtFADs*, *OsFADs*, and *PtFADs* were dominantly enriched in molecular function and biological process.

Table S1. The obtained *PtFAD* sequences using Pfam and BlastP. A total of 23 *PtFAD* family members were identified from the *P. trichocarpa* genome when *Potri.T034100* and *Potri.T180100* localized on the scaffolds, and *Potri.011G137600* and *Potri.018G107700* endowed with the incomplete sequences were eliminated from *PtFAD* candidates.

Table S2. The accession number of *PtFAD*, *AtFAD*, and *OsFAD* members.

Table S3. Summary of *PtFAD* gene family members.

Table S4. Ka/Ks analysis of the *PtFAD* gene pairs. The sequences represented *PtFAD* gene pairs, Ka, Ks, and Ka/Ks indicated the values of Ka (non-synonymous), Ks (synonymous), and Ka/Ks, respectively.

Table S5. Syntenic gene pairs among poplar, Arabidopsis, rice, and willow.

Table S6. The statistics of exons and introns of *PtFAD* and *AtFAD* genes.

Table S7. The e-value, sites, width, sequence, and function of the conserved motifs.

Table S8. The primers used in this study.
